# Safety and Efficacy of Nusinersen Focusing on Renal and Hematological Parameters in Spinal Muscular Atrophy

**DOI:** 10.1002/brb3.70221

**Published:** 2025-01-19

**Authors:** Hüseyin Bahadır Şenol, Gizem Yıldız, Ayşe İpek Polat, Adem Aydın, Ayşe Semra Hız, Alper Soylu, Uluç Yiş

**Affiliations:** ^1^ Department of Pediatric Neurology Dokuz Eylul University Faculty of Medicine İzmir Turkey; ^2^ Department of Pediatric Nephrology Denizli State Hospital Denizli Turkey

**Keywords:** antisense oligonucleotide, spinal muscular atrophy, urine creatinine, urine protein

## Abstract

**Background:**

Spinal muscular atrophy (SMA) is a motor neuron disease caused by mutations in the *SMN1* gene. Nusinersen, an antisense oligonucleotide, has been shown to improve motor function in SMA patients. However, concerns regarding its renal safety remain as previous studies have linked similar treatments to renal toxicity.

**Objective:**

The aim of this study was to evaluate the effects of the nusinersen treatment on platelet counts and renal functions, specifically urine protein excretion, in SMA patients and to estimate safe urinary protein levels before administration of each intrathecal injection.

**Methods:**

This retrospective study examined data from 33 patients with SMA to assess the effects of nusinersen on motor functions and laboratory parameters including platelet count, serum creatinine, urine protein, and urine creatinine. Measurements were taken at baseline andprior to each maintenance dose, after the completion of four initial loading doses. The baseline values were compared between SMA Type 1 and Type 2 patients, while the changes in these values over time were analyzed within each group.

**Results:**

No significant adverse effects on platelet counts or renal functions were observed. Urine creatinine and protein levels were significantly higher in SMA Type 2 patients compared to SMA Type 1 at baseline; these parameters remained stable in SMA Type 2 but increased significantly after the loading doses in SMA Type 1. Motor function improvements were observed in both groups, with the most significant gains in SMA Type 1 after the loading doses. Thus, improvement in motor functions was associated with increase in urine creatinine.

**Conclusion:**

Nusinersen treatment did not cause significant renal toxicity or affect platelet counts. Urine creatinine levels may serve as a potential biomarker for assessing treatment response in SMA Type 1.

## Graphical abstract

The safety and efficacy of nusinersen treatment in 33 patients with spinal muscular atrophy (SMA) Type 1, Type 2, and Type 3 are presented. Hematological and renal parameters, including urine protein, urine creatinine, and the urine protein‐to‐creatinine ratio, were assessed before each nusinersen injection. The CHOP INTEND scores and urine creatinine levels increased in parallel after nusinersen loading, indicating a positive response to treatment in SMA Type 1. Renal parameters remained stable in Type 2 SMA patients. The urine protein‐to‐creatinine ratio was highlighted as a more reliable tool for monitoring renal toxicity, while urine creatinine was proposed as a potential biomarker for evaluating treatment response.

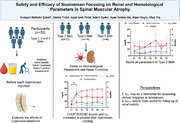

**Abbreviations**
CHOP INTEND: Children's Hospital of Philadelphia Infant Test of Neuromuscular Disorders
HMSFE: Expanded Hammersmith Functional Motor Scale
SCK: serum creatine kinase
S_Cr_
: serum creatinine
SMA: spinal muscular atrophy
*SMN*
: survival motor neuron
*SMN1*
: survival motor neuron 1
*SMN2*
: survival motor neuron 2
U_Cr_
: urine creatinine
U_P_
: urine protein
U_P/Cr_
: urine protein‐to‐creatinine ratio

## 1 Introduction

Spinal muscular atrophy (SMA) is an autosomal recessive motor neuron disease occurring approximately in 1/10,000 births (Sugarman et al. 2012). Deletions or mutations in the survival motor neuron 1 (*SMN1*) gene on chromosome 5q cause a decrease in *SMN1* protein expression, leading to the development of SMA (Kolb and Kissel 2015). Loss of motor neurons in the degenerated anterior horn cells of the spinal cord causes progressive muscle weakness and atrophy resulting in the classical clinical course of the disease (Singh and Singh 2018). The survival motor neuron 2 (*SMN2*) gene is nearly identical to *SMN1*, with only five nucleotide variations. Functional *SMN* protein, although in small amount, can be produced by the *SMN2* mRNA transcript without exon 7 splicing (Oskoui and Servais 2023). Therefore, *SMN2* gene copy number plays a role in determining the phenotype of the disease (Calucho et al. 2018). Nusinersen, an intrathecally administered antisense oligonucleotide, enhances *SMN2* pre‐mRNA splicing to increase the production of *SMN* protein. In December 2016, it was the first disease‐modifying therapy to receive Food and Drug Administration approval for individuals with SMA. The treatment is started with four loading doses in the first 2 months, followed by a maintenance dose every 4 months (*Spinraza (package Insert). Cambridge MBI*, n.d.). Antisense oligonucleotides have been linked to renal toxicity, thrombocytopenia, and coagulation abnormalities (Bennett and Swayze 2010; Chan, Lim, and Wong 2006; Frazier 2015). Therefore, it is recommended to perform a complete blood cell count, quantitative spot urine protein (U_P_) testing, and evaluate coagulation parameters prior to each intrathecal nusinersen injection. A cutoff value for predose platelet count and spot U_P_ level required for treatment was not specified, nor was this information included in the contraindication section of the package insert (*Spinraza (package Insert). Cambridge MBI*, n.d.). The safe treatment profile of nusinersen has been emphasized in previous studies (Goedeker et al. 2021; Stolte et al. 2021). The aim of this study was to evaluate the effects of nusinersen treatment on platelet counts and renal functions, specifically U_P_ excretion, in SMA patients and to estimate safe urinary protein levels before administration of each intrathecal injection. Supporting information

## 2 Methods

### 2.1 Study Design and Participants

A retrospective analysis was conducted on patient files retrieved from the Department of Pediatric Neurology, Dokuz Eylul University Faculty of Medicine, concerning the time interval between September 2017 and January 2024. Data related to age and sex of the patients, type of SMA disease, nusinersen treatment status, age of onset of nusinersen treatment and the number of doses, and laboratory parameters were obtained from patient files (Table 1). Serum creatinine (S_Cr_), creatine kinase (CK), platelets in blood, and protein and creatinine levels in spot urine samples (U_P_ and U_Cr_) were examined at baseline, before each nusinersen injection after four loading doses, along with motor function assessment scores at these time points. As the number of SMA Type 3 patients was limited, comparative and temporal evaluations were made between SMA Type 1 and Type 2 patients.

**TABLE 1:** Characteristics of study subjects.

Features		SMA Type 1 (*n* = 15)	SMA Type 2 (*n* = 14)	SMA Type 3 (*n* = 4)
Gender	male, *n* (%)	5 (33)	9 (64)	1 (25)
	female, *n* (%)	10 (67)	5 (36)	3 (75)
Age at onset of treatment (month)		6.0 (2.16)	68.5 (17.190)	83.5 (26.123)
Treatment	Ongoing	8	14	4
	Number of doses	5 (1.21)	13.5 (5.16)	10 (7.12)
Exitus (*n*/%)		2 (13)	—	—

*Note*: Data are presented as median (min, max) for continuous variables.

Abbreviations: *n*, number of subjects; SMA, spinal muscular atrophy.

### 2.2 Reference Values

The following reference values were used in this study: (1) platelet counts (150–450) × 10^9^/L, (2) normal S_Cr_ (mg/dL) values for term/preterm neonates and older children, proposed by Schwartz et al. (Phadke, Goodyer, and Bitzan 2014; Schwartz, Brion, and Spitzer 1987), (3) in the evaluation of proteinuria, following threshold values were used: (U_P/Cr_): < 0.5 mg/mg for infants aged between 6 and 24 months, and < 0.2 mg/mg for children over 2 years (Hogg et al. 2000). Urinary protein dipstick test threshold values: 1+ protein: 33–67 mg/dL of protein (U_P/Cr_ 0.5–1.0 mg/mg), 2+ protein: 100–200 mg/dL (1.0–4.5 mg/mg), and 3+ protein: > 300 mg/dL (> 4.5 mg/mg) (Lamb, MacKenzie, and Stevens 2009).

### 2.3 Motor Function Assessment

The Children's Hospital of Philadelphia Infant Test of Neuromuscular Disorders (CHOP INTEND) (Glanzman et al. 2010) was utilized to assess the motor function of patients with SMA Type 1. The Expanded Hammersmith Functional Motor Scale (HFMSE) was used to assess the motor function of patients with SMA Type 2 and Type 3 (O'Hagen et al. 2007).

### 2.4 Statistical Evaluation

IBM SPSS statistics version 24.0 (SPSS Inc., Chicago, IL, USA) was utilized for statistical analysis. Frequency distributions were reported as counts and percentages, while continuous variables were expressed as median and range (minimum–maximum). The Shapiro–Wilk test was employed to assess the normality of data distribution. Due to the nonnormal distribution of data, the Mann–Whitney *U* test was used for comparisons of baseline U_P_, U_Cr_, U_P/Cr_, CK, platelet, and S_Cr_ values between the groups. Temporal changes in U_P_, U_Cr_, U_P/Cr_, S_Cr_, platelet values, and motor function scores were analyzed for patients with SMA Types 1 and 2. Median and interquartile range (IQR) values were calculated, and comparisons were made accordingly. Friedman tests were conducted to test whether there is a significant change in the U_P_, U_Cr_, HMSFE, and CHOP INTEND variables due to violations of parametric test assumptions (nonnormal distribution and low number cases, respectively). The Wilcoxon test was performed to test the significance of pairwise differences using the Bonferroni correction to adjust for multiple comparisons. An overall 5% Type 1 error level was used to infer statistical significance. While investigating the associations between nonnormally distributed variables, the correlation coefficients and their significance were calculated using the Spearman test. A 5% Type 1 error level was used to infer statistical significance.

### 2.5 Ethical Publication Statement

The present study was approved by the local ethics committee (number of approval: 2024/07‐15).

## 3 Results

### 3.1 Clinical Characteristics

A total of 33 patients diagnosed with SMA were included in the study. Among them 15 had SMA Type 1, 14 had SMA Type 2, and 4 had SMA Type 3. Two SMA Type 1 patients deceased because of infection. None of these patients experienced any treatment‐related complications except one SMA Type 1 patient who refused treatment after the third dose. All surviving patients completed loading doses. Total doses of nusinersen treatment are shown in Table 1. Side effects did not interrupt the treatment process of any patient.

**FIGURE 1:**
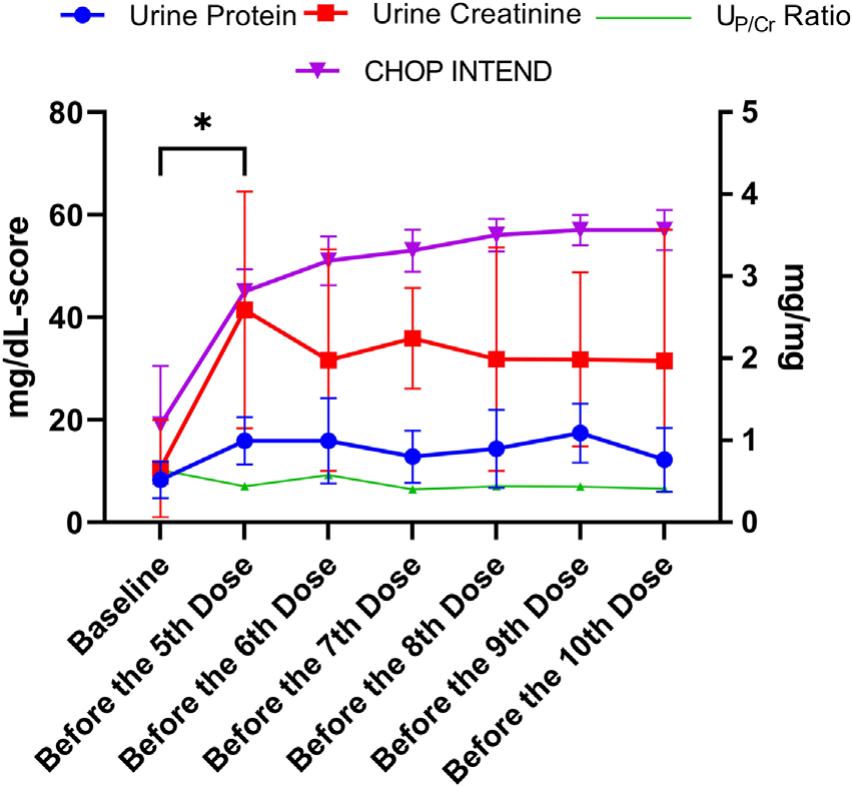
Changes in urine protein, urine creatinine, urine protein‐to‐creatinine ratio, and CHOP INTEND values at different time points in the patients with spinal muscular atrophy Type 1. The units on the left ordinate represent urine protein, urine creatinine, and CHOP INTEND scores, while the unit on the right ordinate corresponds to the urine protein‐to‐creatinine ratio. *: Significant Increase of urine protein, creatinine, and CHOP INTEND values (*p* < 0.05). (For additional data, see Supplementary Data 1.) CHOP INTEND: The Children's Hospital of Philadelphia Infant Test of Neuromuscular Disorders, U_P/Cr_: urine protein‐to‐creatinine ratio.

**FIGURE 2:**
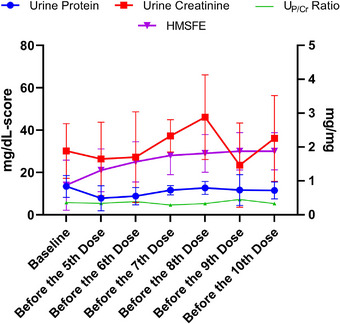
Changes in urine protein, urine creatinine, urine protein‐to‐creatinine ratio, and HMSFE values at different time points in the patients with spinal muscular atrophy Type 2. The units on the left ordinate represent urine protein, urine creatinine, and HMSFE scores, while the unit on the right ordinate corresponds to the urine protein‐to‐creatinine ratio. (For additional data, see Supplementary Data 2.) HMSFE: The Expanded Hammersmith Functional Motor Scale, U_P/Cr_: urine protein‐to‐creatinine ratio.

CHOP INTEND scores in SMA Type 1 patients increased significantly after loading treatment compared to baseline, and this improvement progressively increased with subsequent doses (Figure 1). Similarly, HMSFE scores in SMA Type 2 patients improved significantly with treatment (Figure 2).

### 3.2 Laboratory Indicators

**FIGURE 3:**
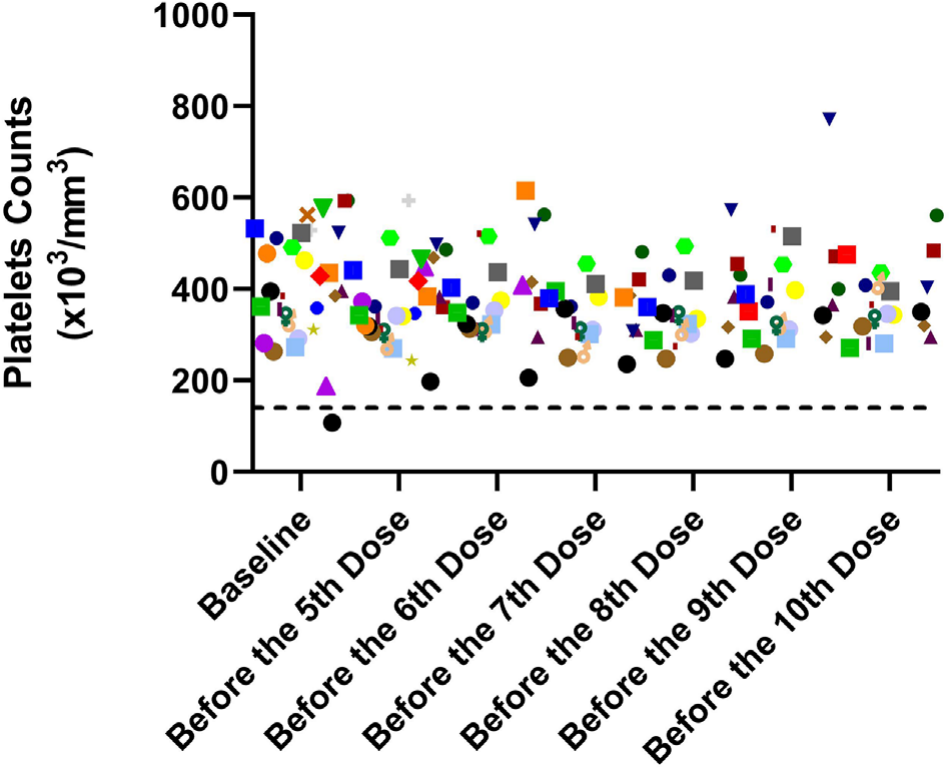
Platelet counts of all patients during treatment (each symbol represents a patient).

Baseline S_Cr_, U_Cr,_ and U_P_ values were found to be higher in patients with SMA Type 2 compared to those with SMA Type 1. Conversely, U_P/Cr_ values were higher in patients with SMA Type 1. Serum creatine kinase (SCK) and platelet values were similar in both groups (Table 2). Predose platelet levels were normal across all three SMA types, except for one patient with baseline thrombocytopenia, which normalized during treatment (Figure 3).

**TABLE 2:** Comparison of biochemical and hematological parameters between SMA Type 1 and Type 2 patients.

Value	SMA Type 1	SMA Type 2	*p*
S_Cr_ (u/L)	**0.115 (0.06)**	**0.150 (0.11)**	**0.031**
SCK (u/L)	132 (93)	122 (70)	0.705
Platelet (×10^3^/mm^3^)	432 (219)	394 (203)	0.164
U_P_ (mg/dL)	**7.450 (8.18)**	**12.450 (9.90)**	**0,039**
U_Cr_ (mg/dL)	**6.535 (12.82)**	**31.5 (29.18)**	**< 0.001**
U_P/Cr_(mg/mg)	**0.585 (0.46)**	**0.350 (0.20)**	**0.012**

*Note*: Statistical analysis was performed using the Mann–Whitney *U* test. Data are presented as median (IQR).

Abbreviations: SCK, serum creatine kinase; S_Cr_, serum creatinine; SMA, spinal muscular atrophy; U_Cr_, urine creatinine; U_P_, urine protein; U_P/Cr_, urine protein‐to‐creatinine ratio.

No significant differences were observed in platelet count, S_Cr_, and U_P/Cr_ during treatment compared to baseline in patients with SMA Types 1 (Table 3) and SMA Type 2 2 (Table 4).

**TABLE 3:** Temporal changes in platelet count, urine protein‐to‐creatinine ratio, and serum creatinine levels in patients with SMA Type 1.

	Baseline	Before the 5th dose	Before the 6th dose	Before the 7th dose	Before the 8th dose	Before the 9th dose	Before the 10th dose	*p* ^a^
Platelet counts (×10^3^/mm^3^) Median (IQR)	459.5 (277.5)	426.5 (169.2)	391.5 (273.7)	348.5 (146.0)	407.5 (184.7)	383 (215.0)	377 (189.5)	0.890
Urine protein‐to‐creatinine ratio (mg/mg) Median (IQR)	0.64 (0.89)	0.44 (0.19)	0.57 (0.72)	0.40 (0.21)	0.44 (0.53)	0.43 (0.28)	0.41 (0.50)	0.792
Serum creatinine (mg/dL) Median (IQR)	0.11 (0.07)	0.11 (0.03)	0.14 (0.05)	0.12 (5.97)	0.11 (0.06)	0.14 (0.05)	0.12 (0.07)	0.361

^a^ Friedman test.

**TABLE 4:** Temporal changes in platelet count, urine protein‐to‐creatinine ratio, and serum creatinine levels in patients with SMA Type 2.

	Baseline	Before the 5th dose	Before the 6th dose	Before the 7th dose	Before the 8th dose	Before the 9th dose	Before the 10th dose	*p* ^a^
Platelet counts (×10^3^/mm^3^) Median (IQR)	356 (199)	337 (60)	354 (123)	311 (81)	335 (116)	372 (142)	346 (90)	0.137
Urine protein‐to‐creatinine ratio (mg/mg) Median (IQR)	0.360 (0.20)	0.340 (0.17)	0.390 (0.21)	0.290 (0.19)	0.33 (0.23)	0.45 (0.43)	0.33 (0.23)	0.276
Serum creatinine (mg/dL) Median (IQR)	0.140 (0.09)	0.120 (0.05)	0.130 (0.06)	0.120 (0.08)	0.140 (0.06)	0.140 (0.06)	0.150 (0.06)	0.569

^a^ Friedman test.

**FIGURE 4:**
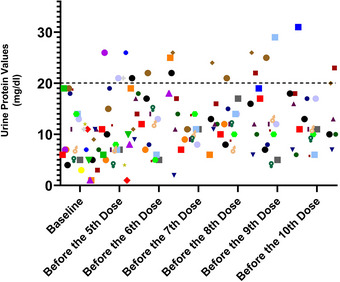
Urine protein values of all patients during treatment (each symbol represents a patient).

Nusinersen was mostly administered when U_P_ levels were below 20 mg/dL, with the highest predose level recorded at 31 mg/dL (Figure 4).

In patients with SMA Type 1, U_P_ and U_Cr_ increased significantly after loading (*p* = 0.039 and *p* = 0.02, respectively) and remained stable thereafter (Figure 1). In contrast, no significant changes in the U_P_ and U_Cr_ were observed during treatment in patients with SMA Type 2 (Figure 2). In SMA Type 1 patients, U_Cr_ values and CHOP INTEND scores were correlated throughout the study (*r* = 0.305, *p* = 0.02).

## 4 Discussion

Nusinersen has affected the clinical course in SMA patients and changed their lives. The efficacy and safety of nusinersen have been confirmed in many studies (Acsadi et al. 2021; Finkel et al. 2017; Lewelt et al. 2015; Mercuri et al. 2018). However, further studies are needed to assess its potential long‐term side effects. In this study, we analyzed the effect of nusinersen treatment on renal function and platelet counts of 33 SMA patients. Unlike previous studies, our study focused on long‐term quantitative data concerning its potential side effects in three types of SMA.

The main concern with nusinersen treatment is the possibility of renal toxicity. Nagarajan et al. (2022) focused on U_P_ levels. The study included 22 patients in the pediatric age group without making any type distinction. Since treatment did not affect protein excretion, they recommended reducing the frequency of U_P_ assessments. In another study, Chen et al. (2023) determined that nusinersen had no negative impact on renal function and U_P_ excretion in 18 pediatric patients with SMA Type 2 and Type 3. Our study yielded results that are consistent with the above‐mentioned two studies in patients with SMA Type 2.

There was a significant increase in U_P_ and U_Cr_ excretion in SMA Type 1 patients after loading doses. Despite the significant rise in U_P_ levels, no renal toxicity was detected, and interruption of the treatment was not required. Recent studies indicate that the greatest motor function improvement during nusinersen treatment is seen following the loading doses (Belančić et al. 2023; Łusakowska et al. 2023). It is also known that patients with SMA Type 1 are the group that benefits the most from nusinersen treatment, showing dramatic improvements in disease progression (Belančić et al. 2023; Yao et al. 2024).

In our study, the period with the most pronounced clinical response in SMA Type 1 patients was also the period with the highest increase in U_Cr_. This may be attributed to the preservation of muscle mass with nusinersen treatment in these patients. In light of these findings, U_Cr_ may serve as a biomarker to evaluate treatment response in SMA Type 1. In addition, U_Cr_ values were significantly higher in SMA Type 2 patients at baseline, who tend to preserve muscle mass more effectively due to the nature of the disease. This further supports the potential of U_Cr_ as a biomarker for evaluating treatment response in SMA Type 1.

Although U_P_ increased in SMA Type 1 patients, the U_P/Cr_ ratio remained stable in all patients during the treatment period. This suggests that nusinersen treatment did not cause a progressive increase in proteinuria. Therefore, U_P/Cr_ ratio determination before nusinersen doses is more functional than U_P_ determination.

Thrombocytopenia is another side effect in clinical studies (Lucas 2018). It has been shown that platelet counts decreased significantly 2 days following nusinersen injections, but increased to pretreatment levels before the next dose (Chen et al. 2023). Nagarajan et al. (2022) stated that platelet counts decreased significantly 4 weeks after initiation of nusinersen treatment. In our study, platelet counts remained stable during the treatment.

Our study had several limitations. The retrospective design, the single‐center experience, and the small patient population were the main limitations of the study. Future studies conducted with a larger, multicenter cohort could make our findings more meaningful.

## 5 Conclusions

No significant adverse effect of nusinersen was detected on platelet counts and renal functions. The U_P/Cr_ ratio, which remained stable during treatment, was more functional in assessing renal toxicity than U_P_ determination only. In SMA Type 1 patients, U_Cr_ values and CHOP INTEND scores were correlated throughout the study. Thus, U_Cr_ may serve as a biomarker to evaluate treatment response in SMA Type 1.

## Author Contributions

**Hüseyin Bahadır Şenol**: conceptualization, investigation, writing–review and editing, writing–original draft, project administration, formal analysis, software. **Gizem Yıldız**: investigation, writing–review and editing, formal analysis. **Ayşe İpek Polat**: conceptualization, investigation, funding acquisition, methodology, formal analysis. **Adem Aydın**: conceptualization, visualization, methodology, data curation, resources. **Ayşe Semra Hız**: data curation, resources, validation, writing–review and editing, software. **Alper Soylu**: conceptualization, writing–original draft, writing–review and editing, supervision. **Uluç Yiş**: conceptualization, writing–original draft, writing–review and editing, investigation, supervision, resources, formal analysis.

## Ethics Statement

The present study was approved by the local ethics committee (number of approval: 2024/07‐15).

## Conflicts of Interest

The authors declare no conflicts of interest.

### Peer Review

The peer review history for this article is available at https://publons.com/publon/10.1002/brb3.70221

## Supporting information



Supporting Information

Supporting Information

## Data Availability

The data that support the findings of this study are available on request from the corresponding author. The data are not publicly available due to privacy or ethical restrictions.
