# Supplementary material for: Safety and Efficacy of Nusinersen Focusing on Renal and Hematological Parameters in Spinal Muscular Atrophy
Source: Brain Behav. 2025 Jan 19;15(1):e70221. doi: 10.1002/brb3.70221 (PMC11743982; doi:10.1002/brb3.70221)
Supplement: Supplementary file 1 — Supporting Information [file BRB3-15-e70221-s001.docx]

Supplementary Data 1: Table of Changes in Urine Protein, Urine Creatinine, and CHOP INTEND Values at Different Time Points in The Patients with Spinal Muscular Atrophy Type 1

|  | Urine Protein (mg/dL) | | Urine Creatinine (mg/dL) | | CHOP INTEND | |
| --- | --- | --- | --- | --- | --- | --- |
|  | Median (IQR) | *p* value | Median (IQR) | *p* value | Median (IQR) | *p* value |
| T1 (Baseline) | 8.750 (7.00) |  | 6.535 (12.82) |  | 19 (22) |  |
| T2 (Before the 5th Dose) | 15.900 (8.25) | *(0.039) (T1-T2) | 28.500 (33.12) | *(0.02) (T1-T2) | 45 (8) | *(0.03) (T1-T2) |
| T3 (Before the 6th Dose) | 15.900 (13.83) | (0.484) (T2-T3) | 29.455 (33.66) | (0.161) (T2-T3) | 51 (7) | *(0.018) (T2-T3) |
| T4 (Before the 7th Dose) | 12.800 (6.55) | (0.176) (T3-T4) | 35.880 (12.83) | (0.866) (T3-T4) | 53 (7) | *(0.018) (T3-T4) |
| T5 (Before the 8th Dose) | 14.350 (13.95) | (0.588) (T4-T5) | 31.850 (30.48) | (0.893) (T4-T5) | 56 (5) | *(0.023) (T4-T5) |
| T6 (Before the 9th Dose) | 17.400 (11.25) | (0.600) (T5-T6) | 31.780 (33.69) | (0.917) (T5-T6) | 57 (5) | *(0.024) (T5-T6) |
| T7 (Before the 10th Dose) | 12.200 (11.33) | (0.753) (T6-T7) | 31.520 (45.70) | (0.345) (T6-T7) | 57 (7) | (0.157) (T6-T7) |

IQR: interquartile range, T: time points, CHOP INTEND: The Children’s Hospital of Philadelphia Infant Test of Neuromuscular Disorders

Pairwise comparisons between consecutive time points were performed using the Wilcoxon rank test. Statistical significance was defined as p<0.05, with significant p-values denoted by (*).
