# Supplementary material for: Safety and Efficacy of Nusinersen Focusing on Renal and Hematological Parameters in Spinal Muscular Atrophy
Source: Brain Behav. 2025 Jan 19;15(1):e70221. doi: 10.1002/brb3.70221 (PMC11743982; doi:10.1002/brb3.70221)
Supplement: Supplementary file 2 — Supporting Information [file BRB3-15-e70221-s002.docx]

Supplementary Data 2: Table of Changes in Urine Protein, Urine Creatinine, and CHOP INTEND Values at Different Time Points in The Patients with Spinal Muscular Atrophy Type 2

|  | Urine Protein (mg/dL) | | Urine Creatine (mg/dL) | | HMSFE | |
| --- | --- | --- | --- | --- | --- | --- |
|  | Median (IQR) | *p* value | Median (IQR) | *p* value | Median (IQR) | *p* value |
| T1 (Baseline) | 13.400 (9.75) |  | 30.150 (23.58) |  | 14 (9) |  |
| T2 (Before the 5th Dose) | 7.800 (8.50) | 0.551 (T1-T2) | 26.390 (34.09) | 0.638 (T1-T2) | 21 (12) | *(0.01) (T1-T2) |
| T3 (Before the 6th Dose) | 8.800 (8.10) | 0.875 (T2-T3) | 27.240 (39.36) | 0.875 (T2-T3) | 25 (9) | *(0.012) (T2-T3) |
| T4 (Before the 7th Dose) | 11.600 (3.85) | 0.657 (T3-T4) | 37.240 (10.77) | 0.594 (T3-T4) | 28 (9) | *(0.002) (T3-T4) |
| T5 (Before the 8th Dose) | 12.700 (4.84) | 0.155 (T4-T5) | 46.100 (33.57) | 0.328 (T4-T5) | 29 (9) | *(0.230) (T4-T5) |
| T6 (Before the 9th Dose) | 11.700 (5.80) | 0.722 (T5-T6) | 23.450 (22.57) | 0.534 (T5-T6) | 30 (11) | *(0.010) (T5-T6) |
| T7 (Before the 10th Dose) | 11.500 (6.25) 4.022 | 0.445 (T6-T7) | 36.070 (35.63) | 0.139 (T6-T7) | 30 (12) | *(0.317) (T6-T7) |

IQR: interquartile range, T: time points, HMSFE: The Expanded Hammersmith Functional Motor Scale

Pairwise comparisons between consecutive time points were performed using the Wilcoxon rank test. Statistical significance was defined as p<0.05, with significant p-values denoted by (*).
